# Eating during the Hemodialysis Session: A Practice Improving Nutritional Status or a Risk Factor for Intradialytic Hypotension and Reduced Dialysis Adequacy?

**DOI:** 10.3390/nu12061703

**Published:** 2020-06-06

**Authors:** Eleni Fotiadou, Panagiotis I. Georgianos, Michail Chourdakis, Pantelis E. Zebekakis, Vassilios Liakopoulos

**Affiliations:** 1 Division of Nephrology and Hypertension, 1st Department of Medicine, AHEPA Hospital, Medical School, Aristotle University of Thessaloniki, St. Kyriakidi 1, GR54636 Thessaloniki, Greece; elena_fotiadou@yahoo.gr (E.F.); pangeorgi@yahoo.gr (P.I.G.); pzempeka@auth.gr (P.E.Z.); 2 Laboratory of Hygiene, Social & Preventive Medicine and Medical Statistics, Medical School, Aristotle University of Thessaloniki, GR54006 Thessaloniki, Greece; mhourd@gapps.auth.gr

**Keywords:** dialysis adequacy, clinical outcomes, hemodialysis, hypotension, nutritional status

## Abstract

Historically, eating during the hemodialysis treatment has been associated with increased risk for adverse intradialytic symptoms and events, risks that have resulted in the implementation of restrictive in-center nutrition policies. Recent studies, however, have recorded a shift in clinical practice with a higher proportion of physicians following the view that administration of intradialytic meals and supplements represents a simple and effective approach to enhance caloric intake and improve nutritional status among patients on hemodialysis. This shift towards less restrictive in-center nutrition practices is mainly supported by evidence from observational studies associating intradialytic nutritional supplementation with improvements in protein-energy wasting, inflammatory state, and health-related quality of life. In sharp contrast, earlier and recent interventional studies have documented that feeding during the hemodialysis treatment provokes a rapid postprandial decline in blood pressure and raises the incidence of symptomatic intradialytic hypotension. Furthermore, other studies have shown that postprandial redistribution in intravascular volume and enhanced blood supply to the gastrointestinal circulation may interfere with the adequacy of the delivered hemodialysis. Those who defend the position that intradialytic nutritional support is beneficial do not dispute the physiology of postprandial hemodynamic response, but they argue against its clinical significance. In this article, we provide an overview of studies that explored the effect of eating during the hemodialysis treatment on intradialytic hemodynamic stability and adequacy of the delivered hemodialysis. We reason that these risks have important clinical implications that are not counteracted by anticipated benefits of this strategy on caloric intake and nutritional status.

## 1. Introduction

In-center nutrition practices vary considerably across dialysis facilities around the world. In a 2014 cross-sectional survey, 61 out of 73 (83.5%) dialysis clinics from 6 continents allowed their patients to eat during the hemodialysis treatment, whereas 53 clinics (72.6%) were providing intradialytic meals [1]. However, dialysis facilities in North America, particularly in the USA, followed more restrictive in-center nutrition policies than dialysis units in European and Asian countries [1]. In a subsequent survey of clinic practices and clinician opinions with respect to feeding during hemodialysis within a large dialysis organization in USA, Benner et al. [2] showed that administration of intradialytic meals was not permitted in 28.6% and 22.6% of clinics in 2011 and 2014, respectively. This shift in practice was statistically significant and was explained by a self-reported increase in the focus of clinicians on nutritional status. 

The above-described disparity on the implemented in-center nutrition practices is in line with the discrepancy between guideline recommendations on the benefits and harms of feeding during the hemodialysis treatment. Based mainly on evidence from observational studies suggesting a potential benefit of intradialytic nutritional supplementation on protein-energy wasting [3,4], inflammatory markers [5] and domains of health-related quality of life [6], a 2018 consensus statement of the International Society of Renal Nutrition and Metabolism supported the position that the administration of meals and supplements during hemodialysis should be considered as a standard-of-care practice to improve nutritional status and clinical outcomes [7]. This statement comes in contrast with an earlier 2007 recommendation of the European Best Practice Guideline (EBPG) group that oral food intake during or just before hemodialysis should be avoided as a preventive measure against intradialytic hypotensive episodes [8]. Evidence to support this guidance is provided by interventional studies showing that oral food intake during hemodialysis induces a more rapid postprandial drop in systemic blood pressure (BP), resulting in a higher incidence of symptomatic intradialytic hypotension [9,10,11,12]. Other studies have also provided evidence that postprandial increase in blood supply to the gastrointestinal circulation interferes with the adequacy of the delivered hemodialysis [12,13,14]. 

Published data create an uncertainty whether receiving food during hemodialysis treatment is overall beneficial, as the anticipated benefit of such strategy on nutritional status has to be reckoned in the clinical implications coming with it. The aim of this article was to explore the effect of eating during the hemodialysis treatment on intradialytic hemodynamic stability and hemodialysis adequacy. 

## 2. Intradialytic Hypotension

Those who defend the position that administration of meals and supplements during hemodialysis is beneficial do not actually dispute the physiology of postprandial drop in BP [15,16,17]. Their argument is that this phenomenon predominantly affects only a minority of patients who are susceptible to intradialytic hypotension, questioning in this way the overall clinical relevance of this complication. 

The exact incidence of intradialytic hypotension is difficult to be quantified with precision in the absence of standardized definitions of this complication. According to the 2007 EBPG recommendations, intradialytic hypotension should be defined as a drop in systolic BP ≥20 mmHg or a decline in mean BP of at least 10 mmHg being accompanied by adverse intradialytic symptoms/events or necessitating nursing interventions [8]. However, this rigorous definition has not been consistently adopted in clinical research. In a 2019 meta-analysis of 5 studies (incorporating data from 1694 participants), the prevalence of intradialytic hypotension (as per the EBPG definition) was 10.1% [95% confidence interval (CI): 6.1–16.5%], with prevalence rate ranging from 5.0% to 30.8% across these 5 studies [18]. Using the nadir systolic BP <90 mmHg definition, the prevalence of intradialytic hypotension (across 5 other studies including a total of 13,189 participants) was 11.6% (95% CI: 8.4–15.7%), with the reported prevalence ranging from 6.7% to 17.2% (18). Studies that reported the proportion of participants with frequent hypotensive episodes during hemodialysis could not be inserted in quantitative data synthesis because of an even higher heterogeneity in the way that frequent intradialytic hypotension has been defined [18]. 

This meta-analytic evidence highlights the unmet need for universally accepted definitions as an approach to estimate the real incidence of intradialytic hypotension. However, the burden and the clinical relevance of this complication should not be underestimated. Intradialytic hypotension is far from being a benign manifestation causing uncomfortable symptoms (i.e., nausea, vomiting, cramps, postdialysis fatigue, etch) [19]. Intradialytic hypotension is a rather common and yet serious hemodialysis-related complication that has been associated with increased risk for a variety of serious adverse clinical events, such as vascular access thrombosis, more rapid loss of residual kidney function, future cardiovascular events, and all-cause mortality [19,20,21,22].

### 2.1. Cross-Sectional Studies

In a cross-sectional analysis of 23 hemodialysis patients, Strong et al. [23] explored the association of oral food and fluid intake with the risk of hypotension over 166 hemodialysis treatments. In regression analysis, the higher the amount of calories and fluids consumed during hemodialysis, the higher was the likelihood of hypotension (*p* = 0.003) and more frequent was the need to intervene (use of mannitol) against intradialytic hypotensive episodes (*p* < 0.001). The incidence of intradialytic hypotension was 2-fold higher in participants with oral intake of >200 calories per session than in those who did not eat during hemodialysis. The use of mannitol was also 5-fold higher in those without any fluid intake during hemodialysis [23]. Another study enrolling 126 stable hemodialysis patients, retrospectively evaluated over 3 consecutive hemodialysis treatments the effect of oral food and fluids intake. Intradialytic hypotension (defined as nadir systolic BP <100 mmHg at any time-point during the session) occurred in 20.6% of the hemodialysis treatments [24]. In multivariable-adjusted regression analysis, there was no association between oral food intake and intradialytic hypotension [24]. This lack of association may be attributable to the fact that a significant proportion of participants failed to achieve their prespecified postdialysis weight at the treatments under study (i.e., the ultrafiltration was not maximized). Another potential confounder of this analysis may be the inadequate detection of intradialytic hypotension, since hypotensive episodes were recorded retrospectively and without evaluation of intradialytic symptoms or nursing interventions, as these parameters were not included in the definition of intradialytic hypotension used in this survey [24]. 

### 2.2. Interventional Studies

Since the cross-sectional design of the above studies cannot demonstrate direct cause-and-effect associations, in the following section, we explore evidence from interventional studies that investigated the effect of eating during hemodialysis on intradialytic hemodynamic stability (Table 1). 

In a prospective controlled trial, Sherman et al. [10] evaluated the postprandial hemodynamic response in 9 non-diabetic hemodialysis patients who had no evidence of neuropathy or orthostatic hypotension. In 62 treatments, a standard meal was administered 1.5–2.0 h after the initiation of hemodialysis, whereas in another 63 treatments, hemodialysis was performed without oral food intake. The rate of decline in mean BP was significantly higher during the postprandial period of fed hemodialysis treatments than during the corresponding period of fasting treatments (−14.4 mmHg/h vs. −2.2 mmHg/h, *p* = 0.03) [10]. During the postprandial period, 13 episodes of symptomatic hypotension in 5 patients were detected. During the corresponding period of fasting treatments, there were only 2 episodes of symptomatic hypotension in 1 patient. The difference in the incidence of intradialytic hypotension between fed and fasting hemodialysis sessions was statistically significant [10]. 

In a randomized, cross-over trial, Zoccali et al. [11] evaluated 13 patients on 2 hemodialysis treatments performed at similar ultrafiltration rates 1-week apart. In the first occasion, a standard snack was administered 1 h after the initiation of hemodialysis; in the second occasion, participants were not allowed to eat during hemodialysis. Although systolic BP fell during both hemodialysis treatments, the rate of decline in systolic BP was significantly higher after the administration of snack than during the corresponding period of fasting hemodialysis (−0.29 mmHg/min vs. –0.08 mmHg/min, *p* < 0.01) [11]. Moreover, the incidence of intradialytic hypotension requiring intravenous saline infusion was higher during the snack-hemodialysis than during the fasting hemodialysis (23 hypotensive episodes in 10 patients vs. 12 hypotensive episodes in 6 patients, *p* = 0.025). The postprandial drop in systolic BP was even more pronounced among participants with underlying autonomic neuropathy [11].

Barakat et al. [9] aimed to elucidate the mechanistic background of the postprandial intradialytic drop of BP in a double-blind, randomized, cross-over trial, in which 10 stable hemodialysis patients were studied in 3 different occasions: (i) administration of placebo 1-hour predialysis and no intradialytic meal; (ii) administration of placebo 1-hour predialysis and consumption of a standard meal 1 h after the initiation of hemodialysis; (iii) administration of caffeine (200 mg) 1-hour predialysis and consumption of a standard intradialytic meal. In accordance with prior studies, mean BP fell sooner and more rapidly after the ingestion of intradialytic meal as compared with the corresponding period of fasting hemodialysis treatment (9). The reduction in systemic vascular resistance index in the absence of a compensatory increase in cardiac output was suggested as a plausible mechanistic explanation for the postprandial drop in systemic BP. Administration of caffeine 1-hour predialysis did not attenuate the hemodynamic alterations provoked by the ingestion of the intradialytic meal [9]. 

These early studies (1988–1993) have been criticized because the hemodynamic response to oral food intake was investigated when hemodialysis was being delivered with acetate dialysate solutions and before the introduction of biocompatible membranes, volumetric ultrafiltration, or low-temperature hemodialysis. However, subsequent studies conducted in the era of optimized hemodialysis delivery confirmed the adverse effect of intradialytic meals on hemodynamic stability, as can be seen below. 

In 1998, Shibagaki et al. [25] applied the method of relative blood volume (RBV) monitoring to explore potential postprandial alterations in the distribution of intravascular volume between the systemic and splachnic circulation inducible by the ingestion of meals during hemodialysis. Among 16 hemodialysis patients who received a standard meal in the supine position, a significant increase in the proportional change of RBV was noted between the pre- and postprandial periods (3.24 ± 0.57 %/h vs. 13.99 ± 0.91 %/h, respectively) [25]. This alteration started simultaneously with the ingestion of a meal but was transient, since the RBV rate of change returned to its basal value within 43 ± 3 min. Changes in intravascular volume appeared to be more prominent with the ingestion of an intradialytic meal in the sitting position. Compared with the basal condition (3.14 ± 0.36 %/h), the RBV rate was increased to 28.21 ± 2.14 %/h during the postprandial period; the change in RBV rate was paralleled with a significant drop in BP levels (152/85 vs. 143/79 mmHg, respectively) [25]. 

In another study, 20 non-diabetic hemodialysis patients received a standard meal 45 min after the initiation of a mid-week hemodialysis session [26]. Ingestion of an intradialytic meal resulted in a significant reduction in the rate of change in RBV (0.24 ± 0.10 %/min vs. 0.08 ± 0.07 %/min, *p* = 0.005). The maximal decline in RBV occurred at 16 min after the ingestion of the meal (range: 8–26 min) and the RBV slope returned to its basal value at a mean of 30 min (range 10–40 min). This transient alteration in intravascular blood volume was accompanied by a parallel postprandial decline in mean BP levels (91 ± 19 vs. 86 ± 20 mmHg, *p* = 0.04) [26]. Other hemodynamic parameters assessed via thoracic bioimpendence analysis (e.g., heart rate, cardiac output, and systemic vascular resistance) remained unmodified. This observation was attributed to an opposing hemodynamic effect of intradialytic meals and ultrafiltration. 

In 2017, Borzou et al. [27] evaluated the postprandial BP response in 48 stable hemodialysis patients studied in 2 occasions: (i) in the first session, a standard meal (~350 kcal of energy) was administered 1 h after the initiation of hemodialysis; (ii) in the second, the same meal was administered 2 h after the initiation of hemodialysis. As expected, a drop in systolic and diastolic BP during the postprandial period was observed in both hemodialysis treatments. However, the timing of oral food intake did not modify the rate or the magnitude of postprandial BP response (1st session: −7.1/−4.4 mmHg, *p* < 0.001; 2nd session: −4.6/−3.0 mmHg, *p* < 0.001) [27]. The incidence of adverse gastrointestinal symptoms (i.e., nausea and vomiting) did not differ between the 2 hemodialysis treatments. 

Colson et al. [28] in 2018 tested the hypothesis that the salt content of intradialytic meals may negatively impact intradialytic hemodynamic stability. They reasoned that administration of meals with high salt content during hemodialysis may be an important source of dietary sodium gain, contributing to an increase in the sense of thirst and to a higher interdialytic weight gain (IDWG). This may precipitate the necessity for more aggressive ultrafiltration rates, resulting in an increased incidence of intradialytic hypotension [30]. In an initial phase of 2 months, 40 hemodialysis patients continued to receive a standard snack with a high salt content (2.4 g) during hemodialysis. In the second phase of this study, participants were administered a snack with a lower salt content (1.4 g per session) for another 4 months [28]. Compared with the initial phase, reduction in dietary sodium gain was accompanied by a significant reduction in IDWG during the second phase of the study (2.17 ± 0.98 vs. 2.03 ± 1.0 kg, *p* < 0.001). The incidence of intradialytic hypotensive episodes was also significantly lower during the low-salt than during the high-salt period of the study (3.2% vs. 6.1% of hemodialysis sessions were complicated by hypotensive episodes, respectively) [28]. These results indicate that administration of intradialytic meals with high salt content turns to be a predisposing factor for intradialytic hypotension. 

In the most recent study published in 2020, 12 non-diabetic hemodialysis patients were evaluated in 3 different occasions in a cross-over design: (i) before and after the administration of a meal during a non-hemodialysis day; (ii) before and after the administration of a meal during a regular hemodialysis treatment; (iii) before and after the administration of 2 meals during hemodialysis [29]. During the non-hemodialysis day, there was no postprandial drop in BP. In contrast, during the hemodialysis treatment with oral meal ingestion, a 22% reduction in systolic BP and a 19% reduction in diastolic BP were observed. Although the ingestion of meal outside of hemodialysis had no effect on arterial wave reflection indices assessed via pulse wave analysis, intradialytic administration of the meal induced a 36% reduction in heart rate-adjusted augmentation index (AIx(75)) [29]. These hemodynamic alterations were slightly greater when patients consumed 2 meals during the hemodialysis treatment. Whether the intradialytic reductions in BP or in AIx(75) are directly attributable to the meal intake or to the hemodialysis procedure per se remains uncertain. The study provided comparisons between hemodialysis and non-hemodialysis days and did not directly compare a fed versus a fasting hemodialysis session [29].

## 3. Hemodialysis Adequacy 

Redistribution of blood volume between the systemic and gastrointestinal circulation during the postprandial period may also affect the adequacy of the delivered hemodialysis. This notion is supported by studies summarized in Table 2 and discussed in detail below.

A prospective controlled study compared the adequacy of a mid-week hemodialysis session performed with versus without oral food intake in 14 stable hemodialysis patients (13). Other parameters affecting the adequacy of hemodialysis (i.e., blood flow and dialysate flow rate, dialyzer, ultrafiltration volume, etch) were similar in both occasions [13]. The urea reduction ratio (URR) and Kt/V calculated via the Daugirdas formula were lower during the fed than during the fasting hemodialysis session (URR: 71.5 ± 5.9% vs. 73.5 ± 6.6%, *p* < 0.05; Kt/V: 1.54 vs. 1.65, *p* < 0.05) [13]. 

Following a similar design, Kara et al. [31] studied 25 non-diabetic hemodialysis patients in 2 consecutive mid-week sessions performed with versus without the administration of a standard meal 1 h after the initiation of hemodialysis. Feeding during hemodialysis resulted in a significantly lower URR and Kt/V as compared with fasting hemodialysis treatment (URR: 67.8 ± 6.1% vs. 72.1 ± 6.0%, *p* < 0.001; Kt/V: 1.4 ± 0.2 vs. 1.6 ± 0.2, *p* < 0.001) [31].

Whereas the above studies suggested that intradialytic ingestion of a meal may interfere with the adequacy of the delivered hemodialysis, another study showed that eating before hemodialysis may not exert a negative impact on URR and Kt/V [14]. In this study, 42 stable hemodialysis patients were asked either not to eat any food for a 3-hour-long period before hemodialysis and over the entire session or were given a standard meal that was consumed 2 h before the initiation of hemodialysis. No differences in URR (72.4 ± 5.2% vs. 72.8 ± 4.7%, *p* = 0.35) and in Kt/V (1.56 ± 0.2 vs. 1.58 ± 0.2, *p* = 0.42) were observed between those who ingested a meal and those who were fasting [14]. Subgroup analyses showed that the lack of effect on adequacy parameters was consistent regardless of the age, gender, and diabetic status of participants. Thus, one alternative approach to reassure the coverage of daily caloric requirements without aggravating the risk of insufficient hemodialysis delivery is to advise our patients to eat 2–3 h before the initiation of hemodialysis.

Meal-associated alterations in hemodialysis adequacy were more analytically investigated in a study that applied the method of continuous real-time Kt/V monitoring [32]. In this study, a standard meal was administered 2 h after the initiation of a mid-week session in 40 stable hemodialysis patients. When Kt/V monitoring was based on the optical dialysate UV-absorbance method, the ingestion of meal induced an immediate drop in online Kt/V. The pre-meal rate of change in online Kt/V was 0.33 ± 0.02/h, but within minutes after oral food intake, the rate of change in Kt/V fell to −0.01 ± 0.04/h. The relative reduction in slope of online Kt/V curve was 101.5 ± 50.4% [32]. This postprandial effect was transient, since the rate of change in Kt/V returned to its basal levels (0.32 ± 0.01/h) within a range of 1–5 min after completion of intradialytic meal. In contrast, when adequacy was monitored via the ionic dialysance method, no significant meal-associated alteration in online Kt/V values was detected. The explanation of the authors for this discrepancy was that meal-associated drop in online Kt/V, as measured by the UV-absorbance method, may be only an artifact related to an intermittent increase in UV-absorbing dialysed solutes [32]. However, the acute and transient fall in online Kt/V may also reflect the redistribution of intravascular volume due to a higher postprandial blood supply to the gastrointestinal circulation.

## 4. Malnutrition

The question that arises is whether restriction of intradialytic meals and supplements is a practice that contributes to the development and/or worsening of malnutrition, which is an important risk factor for increased mortality among patients on hemodialysis [33]. The main argument of those opposing restrictive in-center nutrition practices is that a fixed thrice-weekly hemodialysis regimen may interfere with a regular meal pattern and subsequently with the coverage of caloric requirements, since these patients potentially skip 3 meals per week during their scheduled hemodialysis treatments [15,16]. This notion is supported by an earlier analysis of 1901 hemodialysis patients enrolled in the baseline phase of the National Institutes of Health-sponsored Hemodialysis (HEMO) study showing that dietary energy and protein intake was significantly lower during the hemodialysis treatment days than during the non-hemodialysis treatment days [34]. In addition, more recent studies that evaluated the glycemic profile of diabetic hemodialysis patients using the method of 48-hour continuous glucose monitoring showed that the mean glucose levels were lower and episodes of asymptomatic hypoglycemia were higher during the hemodialysis treatment days than during the non-hemodialysis treatment days [35,36]. The risk of hemodialysis-induced hypoglycemia is higher with the use of glucose-free dialysate solutions, but hypoglycemic episodes may also occur even with a standard glucose-containing dialysate of 5.5 mmol/L [37]. The removal of plasma insulin by the dialyzer and compensatory release of other hormones in response to hypoglycemia represent the main mechanisms involved in pathogenesis of inappropriate postprandial rise in blood glucose levels after the completion of hemodialysis, a phenomenon known as “hemodialysis-related hyperglycemia” [38]. Thus, administration of intradialytic meals could be a preventive measure against hemodialysis-induced hypoglycemia, normalizing also the glycemic profile over the entire hemodialysis treatment day.

These preliminary observations provided rationale for the design of the Fosrenol (lanthanum carbonate) for Enhancing Dietary Protein Intake in Hypoalbuminemic Dialysis Patients (FrEDI) trial [39]. In this trial, 110 hypoalbuminemic hemodialysis patients were randomized to receive high-protein (50–55 g) intradialytic meals combined with 0.5–1.5 g of the phosphate-binder lanthanum carbonate versus low-protein (<1 g) meals during hemodialysis. The dose of lanthanum carbonate was up-titrated every 2 weeks as necessary in the high-protein meal group, whereas background therapy with other phosphate-binders was continued in both arms. Over the 8-week long follow-up, 27% of participants in the high-protein (*n* = 15) versus only 12% of participants (*n* = 6) in the low-protein meal group achieved the primary composite outcome of increase in serum albumin levels of ≥0.2 g/dL while maintaining a target serum phosphate range of 3.5–5.5 mg/dL (*p* value = 0.045) [39]. This benefit on nutritional status was paralleled with an improvement in inflammatory state, since only 9% of participants in the high-protein versus 31% in the low-protein meal group experienced a significant increment in interleukin-6 levels (*p* value = 0.009) [39]. 

Based on results from the FrEDI trial (37), high-protein meals consumed during the hemodialysis treatment in parallel with supervised administration of phosphate-binding agents appears to be a simple and effective strategy to improve protein-energy wasting without aggravating the risk of hyperphosphatemia. It has to be noted, however, that these results should be interpreted within the context of methodological limitations of the FrEDI trial: (i) the inclusion of hypoalbuminemic patients (defined as those with serum albumin <4.0 g/dL) is not necessarily an indication that these patients were malnourished [40] or in need of intradialytic nutritional support. By contrast, the median body mass index (BMI) of participants at baseline was 27.7 kg/m^2^ and the median baseline level of serum phosphorous was 4.9 mg/dL [39], implying that a high proportion of patients enrolled in the FrEDI trial were overweight or obese and had suboptimal phosphate control. Allowing these patients to eat during the hemodialysis treatment may not confer nutritional benefits. (ii) The improvement in serum albumin levels in the high-protein meal group was not accompanied by the anticipated improvement in other more reliable markers of dietary protein intake (such as, serum pre-albumin levels), a finding that makes the results of the FrEDI trial internally inconsistent. (iii) There was a lack of serious adverse events (i.e., mortal events, allergic reactions, or aspiration), but the incidence of intradialytic hypotension or adequacy of the delivered hemodialysis were not recorded even as secondary safety endpoints within the frame of the FrEDI trial [39].

An updated 2018 meta-analysis of 15 randomized trials (incorporating data from 589 participants) aimed to elucidate the benefits and harms of oral nutritional supplements among patients on hemodialysis [41]. Compared with control groups that received placebo or usual care, oral nutritional supplementation was once again associated with a modest improvement in serum albumin levels [weighted mean difference (WMD): 1.58 g/L; 95% CI: 0.52–2.63], as well as with a slight increment in BMI (WMD: 0.40 kg/m^2^; 95% CI: 0.10–0.71). This intervention did not aggravate the risks of hyperphosphatemia and hyperkalemia; no significant between-group difference in serum phosphorus (WMD: −0.17 mg/dL; 95% CI: −0.57 to 0.22) and potassium levels (WMD: 0.06 mEq/L; 95% CI: −0.25 to 0.38) was observed [41]. Whether this modest improvement on nutritional status is translated into a net clinical benefit remains unclear, since none of the trials included in this meta-analysis reported treatment effects of oral nutritional supplementation on “hard” clinical outcomes. Notably, this meta-analysis found evidence of substantial statistical heterogeneity that was attributed to the low quality of eligible trials. In fact, 9 of the 15 trials did not follow a double-blind design and half of them had a short follow-up period of <3 months [41]. Therefore, the benefits of oral nutritional supplements are based on small randomized clinical trials evaluating intermediate endpoints [42] or on observational data [43].

The above data, however, may not be applicable to hospitalized hemodialysis patients with severe malnutrition. Observational studies showed that among critically ill hemodialysis patients (i.e., after major surgery or hospitalization) who were unable to cover their caloric requirements with oral intake, intradialytic parenteral nutritional supplementation was associated with improvement in serum pre-albumin and albumin levels, protein catabolic rate, and inflammatory markers [44]. Parenteral nutritional support was not associated with adverse intradialytic symptoms and did not affect the adequacy of hemodialysis [44]. These potential benefits warrant further investigation in future randomized trials evaluating “hard” clinical endpoints [45]. 

Therefore, one size may not fit all patients in hemodialysis. Overweight and obese patients with inadequate phosphate control may not gain nutritional benefits from the administration of meals during the hemodialysis treatment. For those with autonomic neuropathy, severe congestive heart failure or other risk factors for intradialytic hypotension, feeding during hemodialysis may exacerbate the incidence of adverse intradialytic events. In contrast, in hemodialysis patients with acute illness or recent hospitalization, intradialytic parenteral nutritional support may be an important intervention against protein-energy wasting.

## 5. Conclusions

The question that we have raised in this article is whether patients should be allowed to eat during the hemodialysis treatment. The answer to the argument that restrictive in-center nutrition practices represent a “lost opportunity” to enhance caloric intake and improve protein-energy wasting is that these potential benefits may be counteracted by the risks of intradialytic hemodynamic instability and less efficient hemodialysis delivery. In the absence of solid clinical-trial evidence to demonstrate a clear benefit of intradialytic nutritional support on “hard” clinical outcomes, we recommend that feeding during hemodialysis should not be a blind practice, but such an approach should be individualized according to the nutritional requirements and risk profile of patients for adverse intradialytic events. This therapeutic approach may even be harmful, particularly in those with diabetic autonomic neuropathy or other risk factors predisposing to symptomatic hypotensive episodes during hemodialysis. For these patients, nutritional counselling should focus on dietary interventions implemented outside of the hemodialysis treatment. In any case, there is a need for better designed studies addressing the effect of intradialytic feeding in the various groups of patients on chronic hemodialysis.

## Figures and Tables

**Table 1:** Clinical studies investigating the effect of feeding during dialysis on intradialytic hemodynamic stability.

Study ID	Year	Patients	Intervention	Main Findings
Sherman et al. [10]	1988	9 non-diabetic dialysis patients without autonomic neuropathy or orthostatic hypotension	Dialysis with vs. without oral food intake (62 vs. 63 sessions, respectively)	Postprandial drop in BP. Higher incidence of IDH with oral food intake.
Zoccali et al. [11]	1989	13 stable dialysis patients	Dialysis with vs. without oral intake of a standard snack	Postprandial drop in BP. Higher incidence of IDH with oral food intake.
Barakat et al. [9]	1993	10 stable dialysis patients	Placebo pre-dialysis and no intradialytic meal vs. placebo pre-dialysis and intradialytic mean vs. caffeine 200 mg pre-dialysis and intradialytic mean	Postprandial drop is SVRI and in BP. Administration of caffeine before dialysis did not prevent postprandial IDH.
Shibagaki et al. [25]	1998	21 dialysis patients without evidence of orthostatic hypotension	Administration of standard meal 1 h after initiation of dialysis	Postprandial reduction in blood volume of large vessels assessed with RBV monitoring. Postprandial hypotension.
Sivalingam et al. [26]	2008	20 stable, non-diabetic dialysis patients	Administration of standard meal 45 min after initiation of dialysis	Postprandial reduction in blood volume of large vessels assessed with RBV monitoring. Postprandial drop in mean BP.
Borzou et al. [27]	2016	48 stable dialysis patients	Oral food intake 1 or 2 h after initiation of dialysis vs. no intradialytic meal	Postprandial drop in BP regardless of the timing of oral food intake. No difference in the incidence of nausea or vomiting.
Colson et al. [28]	2018	40 stable dialysis patients	Intradialytic intake of a snack with high (2.4 g/session) vs. low (1.4 g/session) sodium content	Low dietary sodium intake during dialysis was associated with lower IDWG and fewer episodes of symptomatic IDH.
Svinth-Johansen et al. [29]	2020	12 non-diabetic dialysis patients	Administration of a standard meal during a non-dialysis day vs. intradialytic intake of a standard meal.	Intradialytic reduction in BP and in AIx(75). No postprandial hemodynamic effect during the non-dialysis day.

Abbreviations: AIx(75): heart rate-adjusted augmentation index; BP: blood pressure; IDH: intradialytic hypotension; IDWG: interdialytic weight gain; RBV: relative blood volume; SVRI: systemic vascular resistance index.

**Table 2:** Clinical studies investigating the effect of intradialytic food intake on the adequacy of delivered dialysis.

Study ID	Year	Patients	Intervention	Main Findings
San Juan Miguelsanz et al. [13]	2001	14 stable dialysis patients	Midweek dialysis with vs. without oral food intake	Lower URR and Kt/V with intradialytic food intake. No difference in intradialytic symptoms.
Singri et al. [14]	2004	42 stable dialysis patients	Oral food intake 2 h before dialysis vs. 3-hour fasting before dialysis	No difference in URR and Kt/V.
Kara et al. [31]	2010	25 dialysis patients without diabetic autonomic neuropathy	Midweek dialysis with vs. without oral food intake.	Lower URR and Kt/V with intradialytic food intake.
Müller-Deile et al. [32]	2014	40 stable dialysis without symptomatic CV disease	Administration of a standard meal 2 h after the initiation of dialysis	Postprandial drop in online Kt/V assessed with UV absorbance. No change in online Kt/V with ionic dialysance method.

Abbreviations: CV: cardiovascular; URR: urea reduction ratio.
